# Association of preoperative psoas muscle index with clinical outcomes in surgical esophageal cancer patients: a meta-analysis

**DOI:** 10.1186/s12876-026-04915-1

**Published:** 2026-05-14

**Authors:** Yanli Ji, Li Fu, Lei Wang, Lin Lin, E. Zheng, Wenping Wang, Mei Yang

**Affiliations:** 1 Department of Thoracic Surgery, West China Hospital, Sichuan University/West China School of Nursing, Sichuan University, Chengdu, China; 2 Department of Thoracic Surgery, West China Hospital, Sichuan University, Chengdu, China

**Keywords:** Psoas muscle index, Esophageal cancer, Survival, Complication, Meta-analysis

## Abstract

**Background and purpose:**

The prognostic role of preoperative psoas muscle index (PMI) among esophageal cancer patients undergoing the surgery remains unclear. This study aimed to identify the association of preoperative PMI with postoperative clinical outcomes in surgical esophageal cancer patients.

**Methods:**

The PubMed, Web of Science and EMBASE databases were searched up to March 19, 2026. Primary outcome was the survival including the overall survival (OS) and disease-free survival (DFS). Secondary outcome was the postoperative complication such as the anastomotic leak and pneumonia. Hazard ratio (HR) and odds ratio (OR) with 95% confidence interval (CI) were combined to assess the relationship of PMI with primary and secondary outcomes, respectively.

**Results:**

Seventeen studies with 3813 patients were included. As for primary outcomes, pooled results demonstrated that lower PMI was significantly related to worse OS (HR = 1.72, 95% CI: 1.31–2.26, *P* < 0.001) and DFS (HR = 2.15, 95% CI: 1.46–3.16, *P* < 0.001) and subgroup analyses based on the tumor type and neoadjuvant therapy manifested consistent results. As for secondary outcomes, it was identified that preoperative PMI was associated with the risk of postoperative complication (OR = 1.92, *P* < 0.001), anastomotic leak (OR = 2.08, *P* < 0.001), pneumonia (OR = 2.56, *P* < 0.001), arrhythmia (OR = 1.73, *P* < 0.001), cardiac complication (OR = 2.11, *P* = 0.04), dysphagia (OR = 4.05, *P* = 0.03) and mortality (OR = 2.85, *P* < 0.001).

**Conclusion:**

Preoperative PMI was significantly associated with postoperative clinical outcomes and lower PMI indicated shortened survival and increased risk of complication in esophageal cancer patients undergoing the surgery.

**Supplementary Information:**

The online version contains supplementary material available at 10.1186/s12876-026-04915-1.

## Introduction

Esophageal cancer is a major global health problem and remains one of the most lethal malignancies worldwide [1]. According to recent global cancer estimates, approximately 511,054 new cases and 445,391 deaths from esophageal cancer occurred in 2022, ranking it among the leading causes of cancer-related morbidity and mortality worldwide [2]. Despite continuous advances in multimodal treatment, surgery remains the cornerstone of curative treatment for patients with resectable esophageal cancer. Nevertheless, esophagectomy is a highly invasive procedure that is associated with substantial perioperative risk, and postoperative complications remain common even in high-volume centers. In addition to short-term morbidity, patients undergoing surgery for esophageal cancer still face unfavorable long-term outcomes, including disease recurrence and poor survival [3, 4]. Therefore, identifying reliable preoperative markers for risk stratification and prognostic evaluation is of considerable clinical importance in surgical esophageal cancer patients.

Psoas muscle index (PMI) is an imaging-based body composition parameter that is usually calculated by dividing the cross-sectional area of the bilateral psoas muscles on computed tomography by height squared (cm^2^/m^2^) [5]. As a convenient imaging-derived surrogate for skeletal muscle mass, PMI primarily reflects muscle quantity. Reduced skeletal muscle mass is frequently associated with adverse conditions such as malnutrition, sarcopenia, and frailty; however, these constructs are conceptually distinct. In particular, sarcopenia and frailty are multidimensional syndromes incorporating muscle strength, physical performance, and broader functional domains. Therefore, PMI should be interpreted as an indicator of muscle mass rather than a comprehensive measure of frailty or physiological reserve. In recent years, growing evidence has suggested that a low PMI is associated with adverse clinical outcomes in patients with malignant tumors, including reduced survival, poorer treatment tolerance, and increased postoperative morbidity [6, 7]. However, although body composition has attracted increasing attention in oncologic surgery, the prognostic value of PMI in esophageal cancer patients undergoing surgery has not yet been comprehensively clarified.

Therefore, we conducted the present study to systematically evaluate the association between preoperative PMI and clinical outcomes in surgical esophageal cancer patients.

## Methods

This meta-analysis was performed according to the PRISMA guideline 2020 [8].

### Literature search

The PubMed, Web of Science and EMBASE databases were searched up to March 19, 2026 for available studies with following terms: psoas muscle index, psoas muscle mass index, PMI, esophageal, esophagus, tumor, cancer, neoplasm and carcinoma. Detailed search strategy in the PubMed database was presented in the Supplementary file 1. MeSH terms and free texts were used.

### Study selection criteria

Studies meeting following criteria were included: a. patients were diagnosed with primary esophageal cancer; b. patients received the surgery due to esophageal cancer; c. the PMI was preoperatively calculated at the level of lumbar vertebra, normalized by height, and reported in cm^2^/m^2^; d. the association between PMI and at one of the clinical outcomes including the survival and postoperative complication was explored and corresponding effect sizes including the hazard ratio (HR) and odds ratio (OR) with 95% confidence interval (CI) were reported; e. available full texts.

Studies meeting following criteria were excluded: a. letters, editorials, case reports, animal trials or conference abstract; b. insufficient, overlapped or duplicated data.

### Data collection

We collected following data from included studies: the first author, year, country, sample size, study design, tumor-node-metastasis (TNM) stage, tumor type including the squamous cell carcinoma (SCC), adenocarcinoma and esophageal cancer, history of neoadjuvant therapy including the neoadjuvant chemotherapy and neoadjuvant chemoradiotherapy, cutoff value of PMI endpoint for survival, endpoint for complication, HR, OR and 95% CI.

In this meta-analysis, primary outcomes consisted of the overall survival (OS) and disease-free survival (DFS). Secondary outcomes consisted of the overall complication, anastomotic leak, pneumonia, acute respiratory distress syndrome (ARDS), arrhythmia, cardiac complication, chylothorax, deep vein thrombosis (DVT), pulmonary embolism, dysphagia, incision infection, mortality, pneumothorax, postoperative pulmonary complication (PPC), respiratory complication and sepsis.

### Methodological quality assessment

Quality of included studies was assessed by Newcastle–Ottawa Scale (NOS) score tool and studies with a NOS score > 5 were defined as high-quality studies [9].

Study selection, data extraction and quality assessment were performed independently by two investigators (Yanli Ji and Li Fu). Any discrepancies were resolved through discussion with a third reviewer.

### Statistical analysis

Statistical analyses were performed by STATA (version 17.0) software. Heterogeneity between included studies was evaluated by I^2^ statistics. When significant heterogeneity was detected (I^2^ > 50% or *P* < 0.10), the random-effects model was applied; otherwise, the fixed-effects model was applied. HRs and 95% CIs were combined to evaluate the association of PMI with survival. ORs and 95% CIs were combined to evaluate the association of PMI with risk of postoperative complication. Sensitivity analysis was conducted to detect the sources of heterogeneity and assess the stability of the overall results. Besides, Begg’s funnel plot and Egger’s test were conducted to detect publication bias [10, 11]. When significant publication bias was detected, the trim-and-fill method was further applied [9].

## Results

### Literature selection

**Fig. 1:**
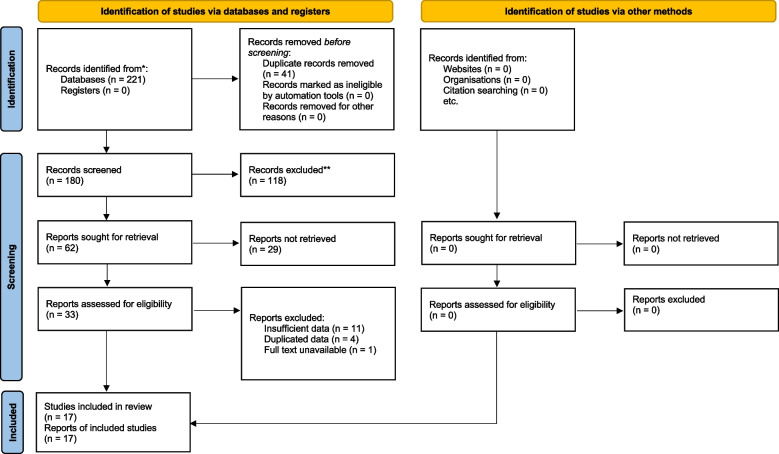
Flow diagram of this meta-analysis

As shown in the Fig. 1., 221 records were searched from databases and 41 duplicated records were removed. After reviewing title, abstracts and full texts, 163 publications were excluded and 17 studies were eventually included [12–28].

### Basic characteristics

Among seventeen included studies, 3813 patients were included with the sample sizes ranged from 63 to 1148. One study was prospective and the other studies were retrospective. All included studies achieved a NOS score greater than 5, indicating generally moderate to good methodological quality according to this assessment tool. Other detailed information was shown in the Table 1.

**Table 1:** Basic characteristics of included studies

Study ID	Country	Sample size	Study design	TNM stage	Tumor type	History of neoadjuvant therapy	Cutoff value of PMI	Endpoint for survival	Endpoint for postoperative complication	NOS
Nagata 2018 [12]	Japan	123	Retrospective	I-IV	NR	Mixed	4.24 cm^2^/m^2^ for men and 2.50 cm^2^/m^2^ for women	NR	Pneumonia	7
Ishida 2019 [13]	Japan	165	Retrospective	I-IV	EC	nCT	6.36 cm^2^/m^2^ for men and 3.92 cm^2^/m^2^ for women	NR	Overall complication, AL, arrhythmia, pneumonia	7
Ozawa 2019 [14]	Japan	82	Retrospective	NR	SCC	nCT or nCRT	5.08 cm^2^/m^2^	DFS	Cardiac complication, PPC	
Kawakita 2020 [15]	Japan	113	Retrospective	II-IIIC	SCC	nCRT	NR	OS, DFS	Overall complication, AL, chylothorax, pneumonia, mortality	7
Mayanagi 2021 [16]	Japan	187	Retrospective	I-IV	EC	nCT	6.36 cm^2^/m^2^ for men and 3.92 cm^2^/m^2^ for women	NR	Overall complication, AL, II, pneumonia	7
Nakayama 2021 [17]	Japan	63	Retrospective	II-III	EC	nCT or nCRT	6.36 cm^2^/m^2^ for men and 3.92 cm^2^/m^2^ for women	OS, DFS	Overall complication	
Uemura 2021 [18]	Japan	150	Retrospective	II-IV	NR	Mixed	6 cm^2^/m^2^	NR	Overall complication, AL, pneumonia	7
Onishi 2022 [19]	Japan	175	Retrospective	II-III	SCC	nCT	5.89 cm^2^/m^2^ for men and 4.06 cm^2^/m^2^ for women	OS	Overall complication	7
Ichinohe 2023 [20]	Japan	131	Retrospective	II-III	SCC	nCT	4 cm^2^/m^2^	OS	Overall complication, AL, RC	7
Ito 2023 [21]	Japan	100	Retrospective	II-III	SCC	nCT	6.11 cm^2^/m^2^ for men and 3.65 cm^2^/m^2^ for women	OS	NR	6
Kitajima 2023 [22]	Japan	150	Retrospective	NR	EC	Mixed	NR	OS, DFS	NR	7
Tian 2023 [23]	China	1148	Retrospective	NR	SCC	No	5.24 cm^2^/m^2^ for men and 3.85 cm^2^/m^2^ for women	NR	AL, ARDS, arrhythmia, pneumonia, 30-day mortality	9
Zhang 2023 [24]	China	356	Retrospective	NR	EC	No	6.36 cm^2^/m^2^ for men and 3.92 cm^2^/m^2^ for women	OS	Overall complication, AL, arrhythmia, pneumonia, 30-day mortality	8
Mann 2024 [25]	Germany	318	Prospective	NR	EC	Mixed	5.3 cm^2^/m^2^ for men and 3.6 cm^2^/m^2^ for women	OS	Overall complication, AL, cardiac complication, PPC, pneumonia, 90-day mortality	
Parvataneni 2024 [26]	India	68	Retrospective	NR	NR	No	5.92 cm^2^/m^2^ for men and 4.00 cm^2^/m^2^ for women	NR	Overall complication, II, RC	
Tan 2024 [27]	*Malaysia*	146	Retrospective	NR	EC	Mixed	4.43 cm^2^/m^2^ for men and 3.26 cm^2^/m^2^ for women	NR	Overall complication, AL, chylothorax, 30-day mortality	7
Aiolfi 2026 [28]	Italy	338	Retrospective	I-IV	AC	Mixed	5.3 cm^2^/m^2^ for men and 3.6 cm^2^/m^2^ for women	OS	Overall complication, AL, ARDS, arrhythmia, chylothorax, DVT/PE, II, pneumonia, pneumothorax, sepsis, 90-day mortality	8

*TNM* Tumor-node-metastasis, *PMI* Psoas muscle index, *NR* Nor reported, *SCC* Squamous cell carcinoma, *AC* Adenocarcinoma, *EC* Esophageal cancer, *nCT* neoadjuvant chemotherapy, *nCRT* neoadjuvant chemoradiotherapy, *OS* Overall survival, *DFS* Disease-free survival, *AL* Anastomotic leak, *ARDS* Acute respiratory distress syndrome, *DVT* Deep vein thrombosis, *PE* Pulmonary embolism, *RC* Respiratory complication, *II* Incision infection; PPC: postoperative pulmonary complication; NOS: Newcastle–Ottawa Scale

### Association of preoperative PMI with survival in surgical esophageal cancer patients

**Fig. 2:**
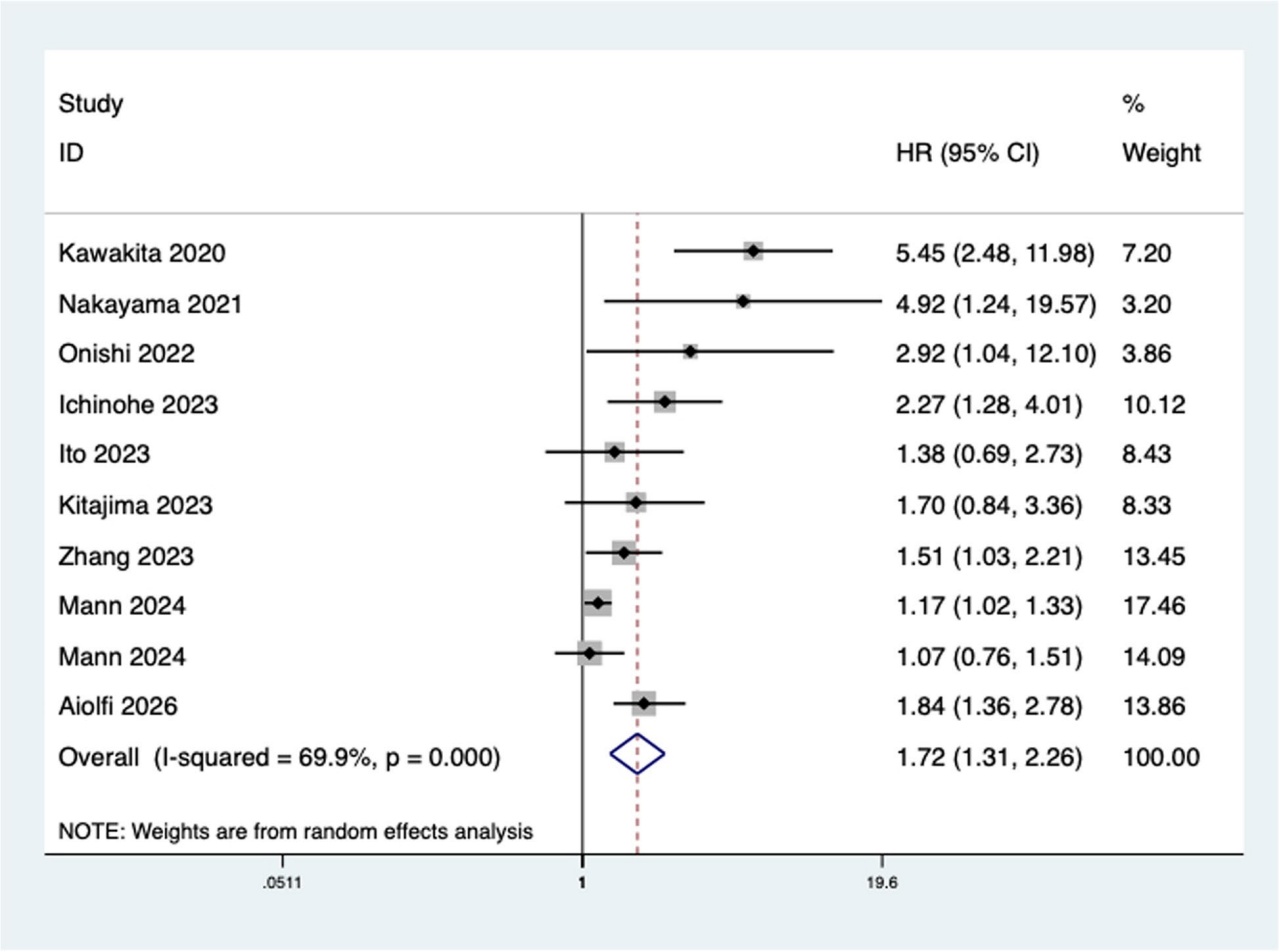
Association of preoperative psoas muscle index with overall survival among surgical esophageal cancer patients

**Fig. 3:**
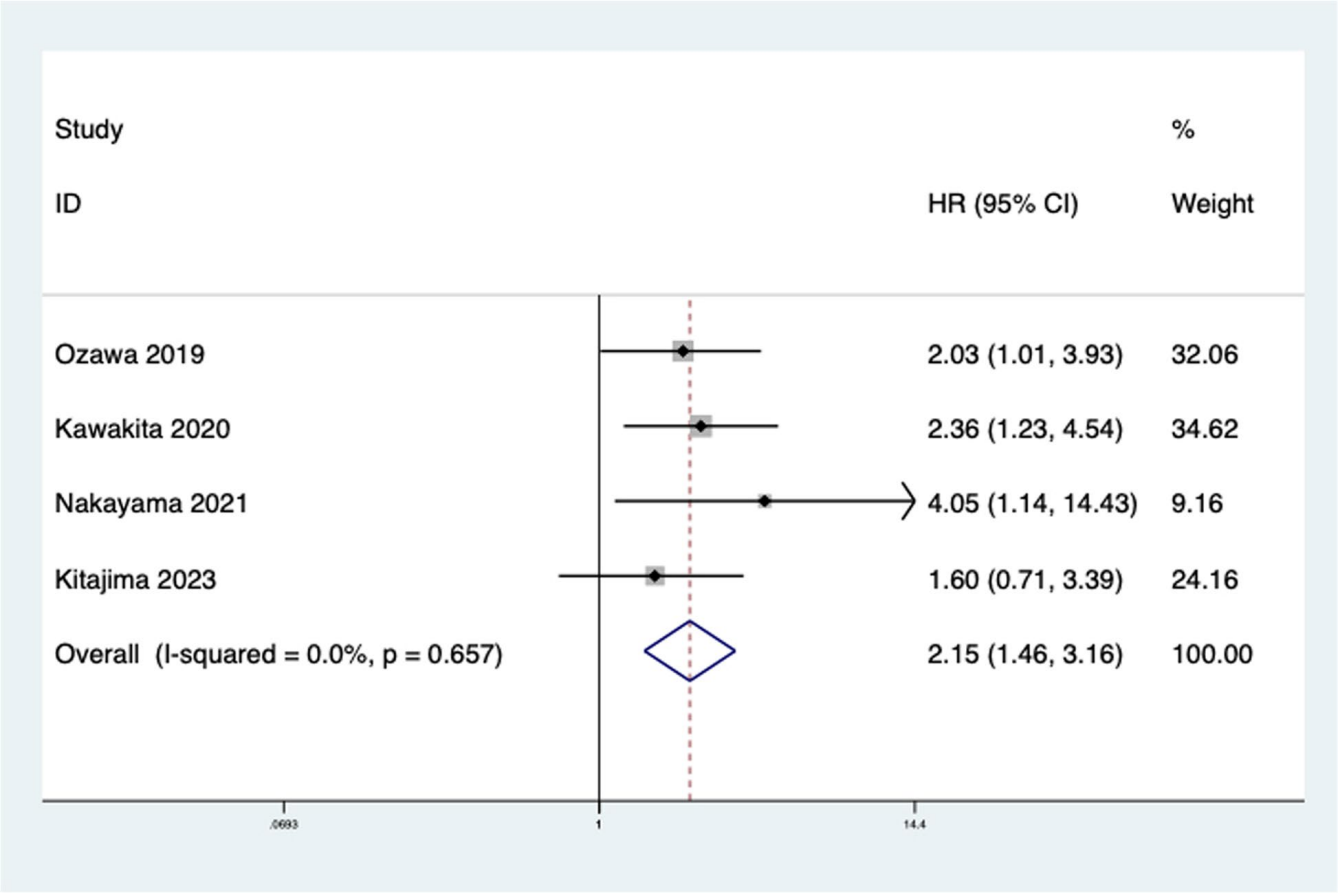
Association of preoperative psoas muscle index with disease-free survival among surgical esophageal cancer patients

Ten studies explored the prognostic role of preoperative PMI with survival among surgical esophageal cancer patients. Pooled results demonstrated that lower PMI was significantly associated with worse OS (HR = 1.72, 95% CI: 1.31–2.26, *P* < 0.001; I^2^ = 69.9%, *P* < 0.001) (Fig. 2) and DFS (HR = 2.15, 95% CI: 1.46–3.16, *P* < 0.001; I^2^ = 0.0%, *P* = 0.657) (Fig. 3). Besides, subgroup analyses based on the tumor type (SCC: HR = 2.55, 95% CI: 1.42–4.56, *P* = 0.002, adenocarcinoma: HR = 1.84, 95% CI: 1.29–2.63, *P* = 0.001; esophageal cancer: HR = 1.30, 95% CI:1.03–1.63, *P* = 0.025) (Supplementary Fig. 1 A) and history of neoadjuvant therapy (yes: HR = 2.73, 95% CI: 1.61–4.63, *P* < 0.001; no: HR = 1.51, 95% CI: 1.03–2.21, *P* = 0.033; mixed: HR = 1.32, 95% CI: 1.03–1.69, *P* = 0.027) (Supplementary Fig. 1B) manifested consistent results (Table 2).

**Table 2:** Results of meta-analysis for primary outcomes

Items	Number of studies	Hazard ratio	95% confidence interval	*P* value	I^2^ (%)	*P* value
Overall survival	9	1.72	1.31–2.26	< 0.001	69.9	< 0.001
Tumor type						
Squamous cell carcinoma	4	2.55	1.42–4.56	0.002	56.1	0.078
Adenocarcinoma	1	1.84	1.29–2.63	0.001	-	-
Esophageal cancer	4	1.30	1.03–1.63	0.025	42.4	0.139
History of neoadjuvant therapy						
Yes	5	2.73	1.61–4.63	< 0.001	48.5	0.101
No	1	1.51	1.03–2.21	0.033	-	-
Mixed	3	1.32	1.03–1.69	0.027	56.5	0.075
Disease-free survival	4	2.15	1.46–3.16	< 0.001	0.0	0.657

### Association of preoperative PMI with risk of complication in surgical esophageal cancer patients

**Fig. 4:**
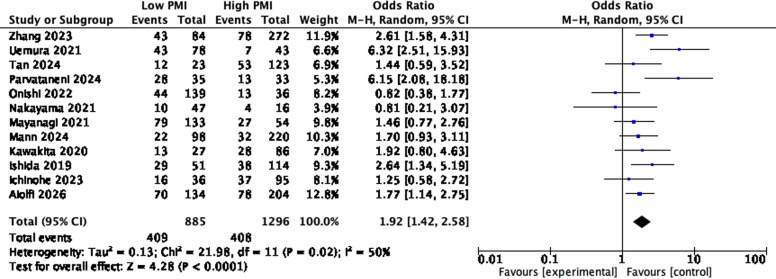
Association of preoperative psoas muscle index with risk of postoperative complication among surgical esophageal cancer patients

Fifteen studies explored the relationship of preoperative PMI with risk of complication among esophageal cancer patients receiving the operation. Pooled results indicated that lower preoperative PMI was associated with increased risk of postoperative overall complication (OR = 1.92, 95% CI: 1.42- 2.58, *P* < 0.001; Fig. 4), anastomotic leak (OR = 2.08, 95% CI: 1.57- 2.75, *P* < 0.001; Supplementary Fig. 2 A), pneumonia (OR = 2.56, 95% CI: 2.10–3.12, *P* < 0.001; Supplementary Fig. 2B), arrhythmia (OR = 1.73, 95% CI: 1.35–2.22, *P* < 0.001; Supplementary Fig. 2 C), cardiac complication (OR = 2.11, 95% CI: 1.02–4.34, *P* = 0.04; Supplementary Fig. 2D), dysphagia (OR = 4.05, 95% CI: 1.14–14.43, *P* = 0.03) and mortality (OR = 2.85, 95% CI: 1.66–4.88, *P* < 0.001; Supplementary Fig. 2E) (Table 3).

**Table 3:** Results of meta-analysis for secondary outcomes

Items	Number of studies	Odds ratio	95% confidence interval	*P* value	I^2^ (%)	*P* value
Overall complication	12	1.92	1.42–2.58	< 0.001	50	0.02
Anastomotic leak	10	2.08	1.57–2.75	< 0.001	0	0.44
Pneumonia	9	2.56	2.10–3.12	< 0.001	0	0.70
Acute respiratory distress syndrome	2	2.08	0.61–7.06	0.24	62	0.10
Arrhythmia	4	1.73	1.35–2.22	< 0.001	0	0.52
Cardiac complication	2	2.11	1.02–4.34	0.04	41	0.19
Chylothorax	3	1.48	0.45–4.90	0.52	0	0.96
Deep vein thrombosis/pulmonary embolism	1	0.76	0.14–4.20	0.75	-	-
Dysphagia	1	4.05	1.14–14.43	0.03	-	-
Incision infection	3	1.37	0.67–2.82	0.39	5	0.35
Mortality	6	2.85	1.66–4.88	< 0.001	0	0.98
Pneumothorax	1	1.07	0.52–2.21	0.85	-	-
Postoperative pulmonary complication	2	1.14	0.33–3.98	0.83	68	0.08
Respiratory complication	2	2.78	0.41–19.03	0.30	86	0.008
Sepsis	1	1.54	0.44–5.43	0.50	-	-

However, no significant association of preoperative PMI with risk of ARDS (OR = 2.08, 95% CI: 0.61–7.06, *P* = 0.24; Supplementary Fig. 3 A), chylothorax (OR = 1.48, 95% CI: 0.45–4.90, *P* = 0.52; Supplementary Fig. 3B), DVT/pulmonary embolism (OR = 0.76, 95% CI: 0.14–4.20, *P* = 0.75), incision infection (OR = 1.37, 95% CI: 0.67–2.82, *P* = 0.39; Supplementary Fig. 3 C), pneumothorax (OR = 1.07, 95% CI: 0.52–2.21, *P* = 0.85), PPC (OR = 1.14, 95% CI: 0.33–3.98, *P* = 0.83; Supplementary Fig. 3D), respiratory complication (OR = 2.78, 95% CI: 0.41–19.03, *P* = 0.30; Supplementary Fig. 3E) or sepsis (OR = 1.54, 95% CI: 0.44–5.43, *P* = 0.50) was observed (Table 3).

### Sensitivity analysis

**Fig. 5:**
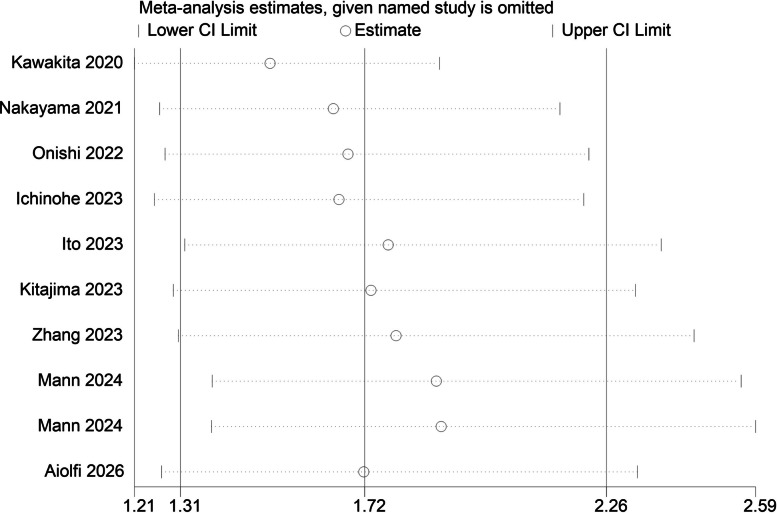
Sensitivity analysis about the association of preoperative psoas muscle index with overall survival among surgical esophageal cancer patients

According to the sensitivity analysis (Fig. 5), the pooled results for OS were stable and reliable and none of the included studies, including the largest study (Tian 2023) or the prospective cohort (Mann 2024), materially altered the pooled effect estimate.

### Publication bias

**Fig. 6:**
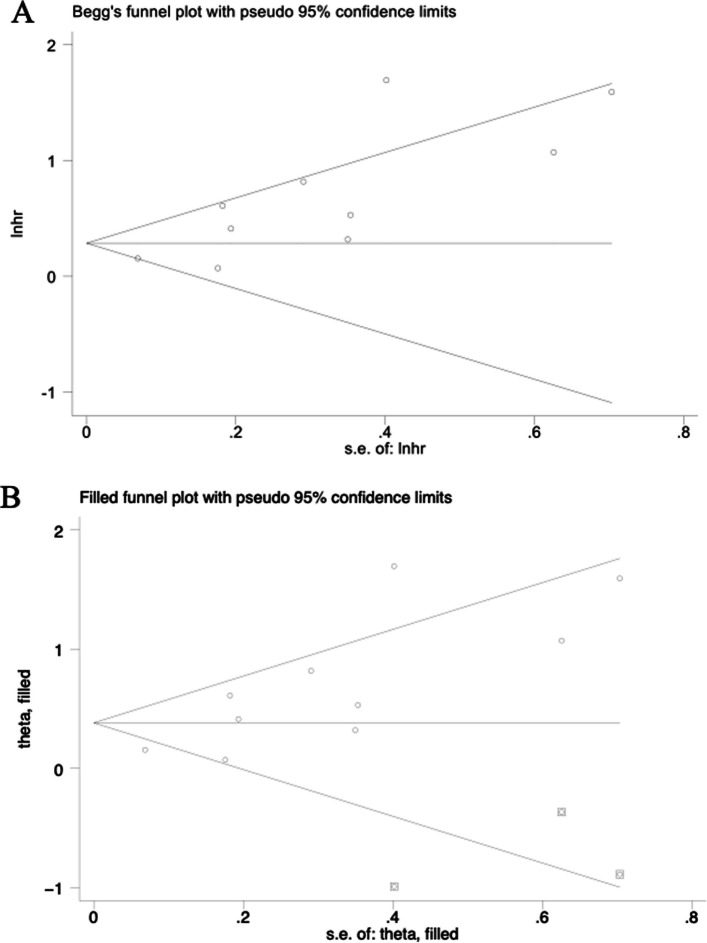
Begg’s funnel plot (**A**) and filled funnel plot (**B**) about the association of preoperative psoas muscle index with overall survival among surgical esophageal cancer patients

According to the Begg’s funnel plot (Fig. 6A) and Egger’s test (*P* = 0.007), significant publication bias was detected. Thus, the trim-and-fill method was applied and three potentially unpublished studies were observed (Fig. 6B). However, these three studies did not affect the overall conclusion (fixed HR = 1.29, 95% CI: 1.16–1.43, *P* < 0.001; random HR = 1.46, 95% CI: 1.10–1.94, *P* = 0.008), which also indicate the stability of our findings.

## Discussion

In this meta-analysis, we found that lower preoperative PMI was significantly associated with poorer long-term survival and a higher risk of multiple postoperative adverse outcomes in surgical esophageal cancer patients. Specifically, patients with lower PMI had significantly poorer OS and DFS, together with increased risks of overall postoperative complications, anastomotic leak, pneumonia, and postoperative mortality. Associations with arrhythmia, cardiac complications, and dysphagia were also observed, although these findings were based on a limited number of studies. These findings suggest that PMI, an imaging-derived marker readily available from routine preoperative CT, may provide clinically meaningful information for perioperative risk stratification [29].

Several mechanisms may explain these associations. PMI reflects skeletal muscle mass and may serve as a surrogate marker of nutritional status and physiological reserve. In patients with esophageal cancer, tumor-related dysphagia, systemic inflammation, and treatment-related toxicities may contribute to progressive muscle depletion before surgery, thereby reducing tolerance to major surgical stress and impairing postoperative recovery [7, 25]. Low muscle mass has also been linked to impaired immune function, decreased cardiopulmonary capacity, and reduced tissue repair ability, which may increase susceptibility to complications such as pneumonia and anastomotic leak. Furthermore, diminished muscle reserve may limit tolerance to multimodal oncologic treatment and reduce resilience to disease recurrence, contributing to inferior long-term survival [4, 30, 31].

Importantly, PMI should be interpreted as a marker of muscle quantity rather than a comprehensive measure of sarcopenia or frailty, which require multidimensional assessment including muscle strength and functional performance. Therefore, PMI likely captures one aspect of biological vulnerability and should be considered within a broader clinical context rather than as a standalone determinant of outcome [32, 33].

Although some postoperative outcomes did not reach statistical significance in our pooled analyses, the observed effect directions were still generally unfavorable in patients with low PMI. For example, the pooled ORs for acute respiratory distress syndrome, chylothorax, incision infection, postoperative pulmonary complications, respiratory complications, and sepsis were mostly above 1.0, suggesting a possible clinical trend toward increased risk, even though the confidence intervals crossed the null. This pattern may be attributable to the relatively limited number of studies available for several individual complications, insufficient event numbers, and between-study differences in surgical approach, perioperative management, complication definitions, and PMI cutoff values. Therefore, the lack of statistical significance for some endpoints may be partly attributable to limited study numbers and event counts. However, given the small number of studies contributing to several outcomes, these findings should be interpreted cautiously and require further validation. A similar phenomenon has been reported in recent esophageal surgery literature, where some adverse outcomes failed to reach formal statistical significance despite showing clinically unfavorable trends [34].

From a clinical perspective, PMI may have practical value in the perioperative management of esophageal cancer patients. Because PMI can be obtained from routine preoperative CT without additional invasive procedures or substantial cost, it may serve as a convenient adjunct for preoperative risk stratification. Identification of patients with low PMI may prompt more comprehensive nutritional evaluation, early initiation of nutritional support, exercise-based prehabilitation, respiratory muscle training, and intensified perioperative monitoring [35]. In addition, PMI assessment may assist clinicians in shared decision-making by informing discussions regarding operative risk, expected recovery, and the need for closer postoperative surveillance. However, whether PMI-guided interventions can directly improve clinical outcomes requires further prospective validation [35, 36]. From a translational perspective, the appeal of PMI lies in its accessibility and reproducibility, as it can be derived from routine preoperative imaging without additional cost or patient burden. However, its clinical utility will likely depend on integration with complementary markers, including functional assessments, inflammatory or metabolic biomarkers, and established clinical risk models. Rather than serving as a standalone determinant, PMI may contribute incremental prognostic information within a multidimensional risk stratification framework.

Several limitations of this meta-analysis should be acknowledged. First, most included studies were retrospective in nature, which inevitably introduces selection bias, residual confounding, and limited control over perioperative heterogeneity. Therefore, the observed associations should not be interpreted as causal relationships. Second, the cutoff values and measurement methods used to define low PMI varied across studies, which may have contributed to between-study heterogeneity and reduced the comparability of pooled estimates. In addition, substantial heterogeneity was observed for overall survival (I^2^ = 69.9%). Although subgroup analyses based on tumor type and neoadjuvant therapy showed consistent results, other potential sources of heterogeneity such as tumor stage distribution, surgical approach, geographic differences, and variations in PMI cutoff determination could not be further explored due to limited available data. Third, because original individual-level data were unavailable, we were unable to perform more detailed subgroup analyses according to potentially important modifiers such as age, tumor stage, comorbidity burden, or other clinicopathological characteristics. Fourth, although PMI is a practical imaging-based indicator of muscle mass, it does not capture muscle strength or physical performance and therefore should not be considered equivalent to a formal diagnosis of sarcopenia, which requires multidimensional assessment. Similarly, frailty and nutritional status represent broader clinical constructs encompassing functional, metabolic, and systemic components beyond muscle quantity alone. Thus, caution is warranted when interpreting PMI as a surrogate for these conditions. Fifth, multiple secondary outcomes were analyzed in this meta-analysis without formal adjustment for multiple comparisons. Although these endpoints represent clinically distinct complications rather than repeated testing of a single hypothesis, the possibility of false-positive findings cannot be completely excluded. Therefore, the results regarding secondary outcomes should be interpreted with caution and considered exploratory in nature. Sixth, although HRs and ORs were extracted preferentially from multivariate analyses when available, the adjustment strategies and confounding factors varied across studies, and in a few cases only univariate estimates were reported. Differences in covariate adjustment may have influenced the pooled effect estimates. Due to the large number of outcomes included, detailed adjustment variables for each study were not fully summarized, which should be considered when interpreting the results. Seventh, several secondary outcomes were based on a limited number of studies, and in some cases only one study contributed data. The small sample size and event numbers for these outcomes may reduce statistical precision and increase the risk of unstable or inflated effect estimates. Therefore, these results should be interpreted with particular caution. Eighth, some specific postoperative complications, including arrhythmia, cardiac complications, and dysphagia, were evaluated in only a small number of studies, and in certain cases a single study contributed data. Therefore, these associations should be interpreted cautiously and require confirmation in future large-scale prospective research. Finally, significant publication bias was detected for overall survival (Egger’s test *P* = 0.007), suggesting potential small-study effects. After applying the trim-and-fill method, the pooled hazard ratio was attenuated (HR = 1.46) but remained statistically significant. This finding indicates that although the direction of association appears consistent, the magnitude of the effect in the unadjusted model may have been overestimated. Therefore, the strength of the association should be interpreted cautiously.

## Conclusion

In conclusion, lower preoperative PMI was significantly associated with poorer long-term survival and a higher risk of several postoperative adverse outcomes in surgical esophageal cancer patients. These findings suggest that PMI may serve as a simple and useful imaging-based marker for preoperative risk stratification and perioperative management. However, given the retrospective nature of most included studies and the heterogeneity in PMI cutoff values, further large-scale prospective studies are warranted to confirm its clinical utility.

## Supplementary Information

Supplementary Material 1.

Supplementary Material 2. Supplementary figure 1. Subgroup analysis based on the tumor type (A) and history of neoadjuvant therapy (B) about the association of preoperative psoas muscle index with overall survival among surgical esophageal cancer patients.

Supplementary Material 3. Supplementary figure 2. Association of preoperative psoas muscle index with risk of anastomotic leak (A), pneumonia (B), arrhythmia (C), cardiac complication (D) and mortality (E) among surgical esophageal cancer patients.

Supplementary Material 4. Supplementary figure 3. Association of preoperative psoas muscle index with risk of acute respiratory distress syndrome (A), chylothorax (B), incision infection (C), postoperative pulmonary complication (D) and respiratory complication (E) among surgical esophageal cancer patients.

## Data Availability

All data generated or analyzed during this study are included in this published article.
