# Supplementary figures and images for: Association of preoperative psoas muscle index with clinical outcomes in surgical esophageal cancer patients: a meta-analysis
Source: BMC Gastroenterol. 2026 May 14;26:421. doi: 10.1186/s12876-026-04915-1 (PMC13343671; doi:10.1186/s12876-026-04915-1)

Detailed search strategy in the **PubMed database.**


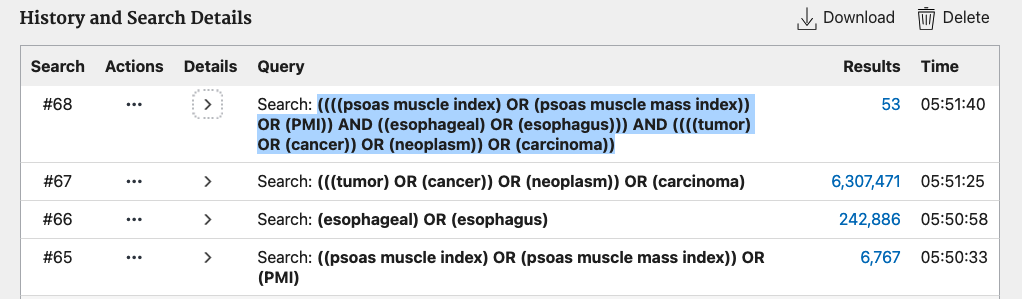

Supplement: Supplementary file 1 — Supplementary Material 1. [file 12876_2026_4915_MOESM1_ESM.docx]
