# Supplementary material for: Association of preoperative psoas muscle index with clinical outcomes in surgical esophageal cancer patients: a meta-analysis
Source: BMC Gastroenterol. 2026 May 14;26:421. doi: 10.1186/s12876-026-04915-1 (PMC13343671; doi:10.1186/s12876-026-04915-1)

Supplementary figure 1A. Subgroup analysis based on the tumor type about the association of preoperative psoas muscle index with overall survival among surgical esophageal cancer patients.


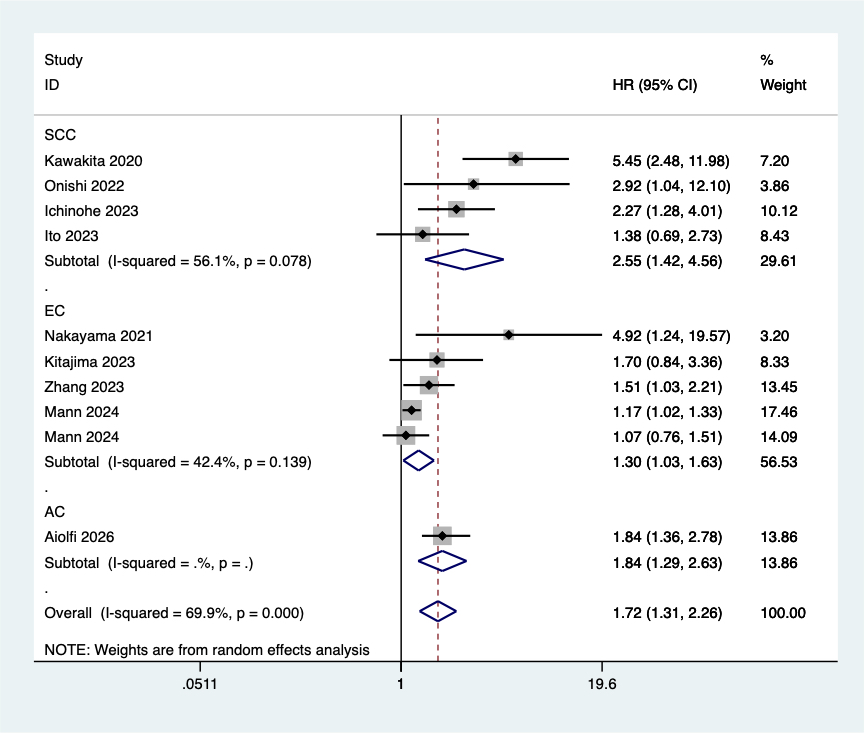


Supplementary figure 1B. Subgroup analysis based on the history of neoadjuvant therapy about the association of preoperative psoas muscle index with overall survival among surgical esophageal cancer patients.


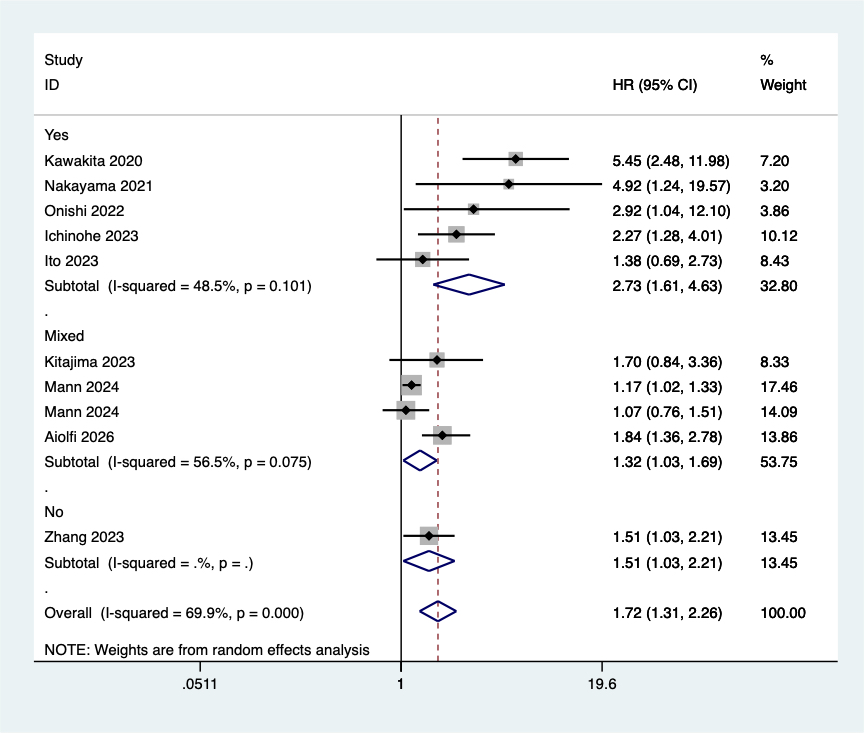

Supplement: Supplementary file 2 — Supplementary Material 2. Supplementary figure 1. Subgroup analysis based on the tumor type (A) and history of neoadjuvant therapy (B) about the association of preoperative psoas muscle index with overall survival among surgical esophageal cancer patients. [file 12876_2026_4915_MOESM2_ESM.docx]
