# Supplementary material for: Association of preoperative psoas muscle index with clinical outcomes in surgical esophageal cancer patients: a meta-analysis
Source: BMC Gastroenterol. 2026 May 14;26:421. doi: 10.1186/s12876-026-04915-1 (PMC13343671; doi:10.1186/s12876-026-04915-1)

Supplementary figure 2A. Association of preoperative psoas muscle index with risk of anastomotic leak among surgical esophageal cancer patients.


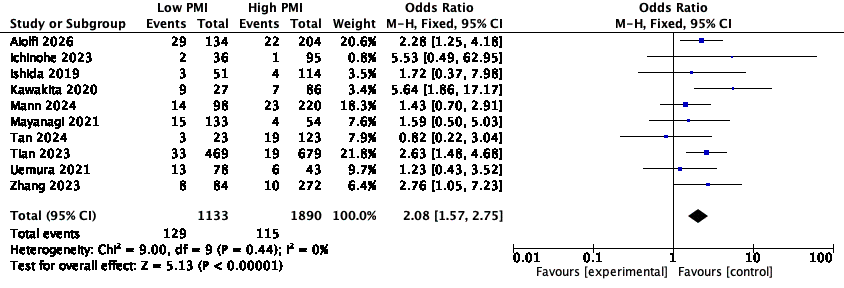


Supplementary figure 2B. Association of preoperative psoas muscle index with risk of pneumonia among surgical esophageal cancer patients.


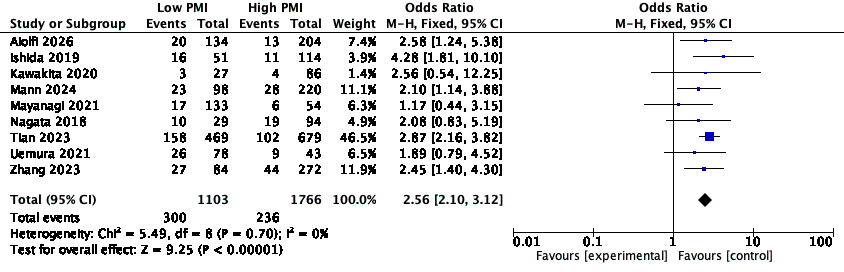


Supplementary figure 2C. Association of preoperative psoas muscle index with risk of arrhythmia among surgical esophageal cancer patients.


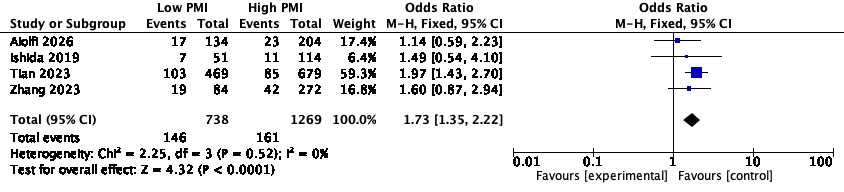


Supplementary figure 2D. Association of preoperative psoas muscle index with risk of cardiac complication among surgical esophageal cancer patients.


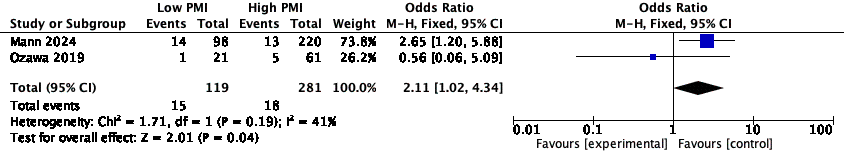


Supplementary figure 2E. Association of preoperative psoas muscle index with risk of mortality among surgical esophageal cancer patients.


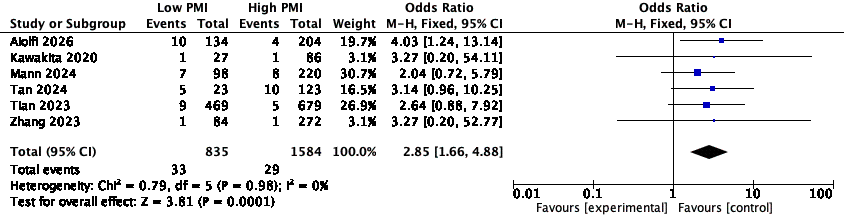

Supplement: Supplementary file 3 — Supplementary Material 3. Supplementary figure 2. Association of preoperative psoas muscle index with risk of anastomotic leak (A), pneumonia (B), arrhythmia (C), cardiac complication (D) and mortality (E) among surgical esophageal cancer patients. [file 12876_2026_4915_MOESM3_ESM.docx]
