# Supplementary material for: Association of preoperative psoas muscle index with clinical outcomes in surgical esophageal cancer patients: a meta-analysis
Source: BMC Gastroenterol. 2026 May 14;26:421. doi: 10.1186/s12876-026-04915-1 (PMC13343671; doi:10.1186/s12876-026-04915-1)

Supplementary figure 3A. Association of preoperative psoas muscle index with risk of acute respiratory distress syndrome among surgical esophageal cancer patients.


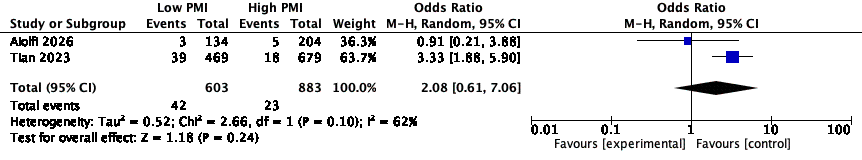


Supplementary figure 3B. Association of preoperative psoas muscle index with risk of chylothorax among surgical esophageal cancer patients.


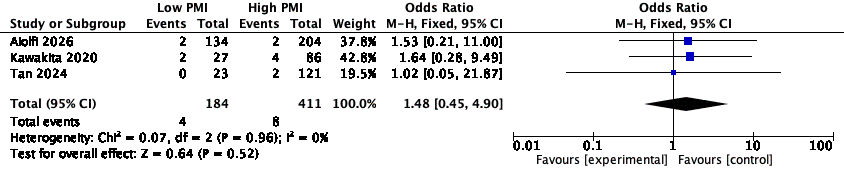


Supplementary figure 3C. Association of preoperative psoas muscle index with risk of incision infection among surgical esophageal cancer patients.


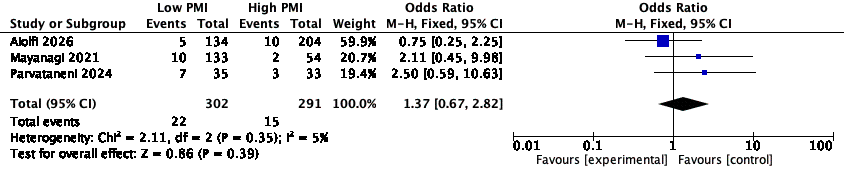


Supplementary figure 3D. Association of preoperative psoas muscle index with risk of postoperative pulmonary complication among surgical esophageal cancer patients.


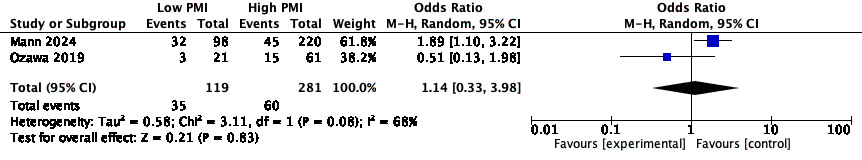


Supplementary figure 3E. Association of preoperative psoas muscle index with risk of respiratory complication among surgical esophageal cancer patients.


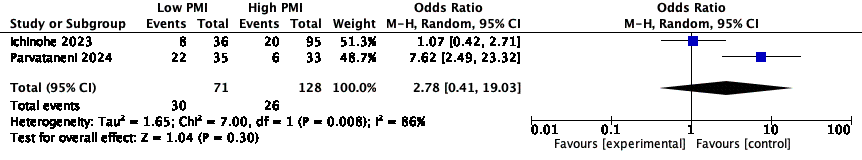

Supplement: Supplementary file 4 — Supplementary Material 4. Supplementary figure 3. Association of preoperative psoas muscle index with risk of acute respiratory distress syndrome (A), chylothorax (B), incision infection (C), postoperative pulmonary complication (D) and respiratory complication (E) among surgical esophageal cancer patients. [file 12876_2026_4915_MOESM4_ESM.docx]
